# Context-aware LLM extraction and controlled-vocabulary normalization of GEO transcriptomic sample metadata

**DOI:** 10.21203/rs.3.rs-10844781/v1

**Published:** 2026-08-31

**Authors:** Mateusz Szczepaniak, Jonathan Wren

**Affiliations:** Oklahoma Medical Research Foundation; Oklahoma Medical Research Foundation

**Keywords:** Gene Expression Omnibus, metadata harmonization, large language models, MeSH, transcriptomics, text mining, automated annotation

## Abstract

**Motivation:**

Public transcriptomic repositories contain millions of samples, but reuse is limited by inconsistent depositor-provided metadata. In the Gene Expression Omnibus (GEO), biologically critical fields such as tissue, disease state and treatment are frequently encoded within heterogeneous free text descriptions, undefined abbreviations/acronyms, or implicit experimental context.

**Results:**

We developed a locally deployable large language model (LLM) pipeline converting GEO sample metadata into structured Tissue, Condition and Treatment labels. It performs sample-level extraction (Phase 1), study-level contextual inference of missing labels from GSE context and sibling-sample label distributions (Phase 1b), and controlled-vocabulary entity-linking, also called concept normalization, to Medical Subject Headings (MeSH) and Cellosaurus cell-line identifiers (Phase 2), with out-of-vocabulary clustering for concepts not within these vocabularies. We evaluated 804,427 human GEO samples against other automated annotations and manually curated benchmarks for Tissue, Condition and Treatment. On the manual benchmark, the extractor labelled Tissue and Condition at 99.67% and 95.0% accuracy. Entity linking reached accuracies of 0.985, 0.993 and 0.982 (F1 = 0.993, 0.996 and 0.991) on the labels for which a controlled-vocabulary answer exists, and separately assigned an identifier at all to 80.69%, 62.91% and 23.13% of extracted labels against 31.68%, 35.01% and 7.46% for deterministic exact MeSH matching.

**Availability:**

All code, evaluation and results are provided as Supplementary Data. An interactive program built from the pipeline is available at https://github.com/SciSpectator/LLM-GEO-Label-Extractor.

## 1 Introduction

Public transcriptomic repositories, such as GEO (Barrett et al. 2013, Rung and Brazma 2013, Clough et al. 2024), are essential to help better understand disease signatures and for expression meta-analysis. However, analysis is frequently limited not by data availability, but by the ability to select the covariates of interest from within the free-text, unstructured, often incomplete depositor metadata. To identify a disease, tissue, treatment, or control cohort for further analysis across studies remains difficult, because these covariates are not in a standard, structured form. These issues directly affect FAIR (Findable, Accessible, Interoperable, Reusable) principles for public biomedical repositories, whereby metadata quality, standardization, and validation affect both discoverability and secondary analysis (Wilkinson et al. 2016, Gonçalves and Musen 2019).

GEO sample records (GSMs) contain rich textual descriptions spread across a title such as “subject_1-Ascending colon-biopsy”, a source name, a characteristics field of key-value pairs, protocol text and a free-text description. These fields are depositor-written and often contain study-specific nomenclature or abbreviations, inconsistent disease names, treatment details embedded in protocols, or implicit context that is only apparent from the parent GEO series (GSE) metadata, which has the same issues (Brazma et al. 2001, Gonçalves and Musen 2019). For example, a disease such as breast cancer may appear as “mammary tumor”, “breast carcinoma”, etc. Tissue and treatment fields suffer from the same problems, but may also use cell-line nomenclature, contain drug names and doses, case/control status, and mix in protocol text with sample-processing details separate from treatment details. This heterogeneity creates a practical barrier to reuse of these datasets for cross-study and/or meta-analyses (Rung and Brazma 2013, Wilkinson et al. 2016, Gonçalves and Musen 2019).

Some existing resources address parts of this problem. NCBI BioSample and EBI BioSamples organize sample metadata across assay types, while GEOmetadb provides structured access to GEO records (Barrett et al. 2012, Gostev et al. 2012, Zhu et al. 2008). More directly, ALE extracts selected GEO sample attributes using heuristic and ontology-based methods (Giles et al. 2017). MetaSRA normalizes SRA BioSample metadata using lexical matching and rule-based reasoning (Bernstein et al. 2017), and CREEDS uses GEO study and sample metadata to identify experimental contrasts and construct expression signatures (Wang et al. 2016). Biomedical language models such as BioBERT and PubMedBERT support named-entity and relation extraction, while SapBERT and BERN2 address biomedical entity recognition and/or normalization (Lee et al. 2020, Gu et al. 2021, Liu et al. 2021, Sung et al. 2022). UMLS, BioPortal and the OBO Foundry provide controlled terminology infrastructure, while MetaMap, cTAKES and PubTator Central map biomedical text to controlled concepts (Bodenreider 2004, Smith et al. 2007, Whetzel et al. 2011, Aronson and Lang 2010, Savova et al. 2010, Wei et al. 2019). These approaches are not designed to treat a GEO sample as part of a structured study in which parent-series metadata and sibling samples can disambiguate sample-specific labels. In contrast, our workflow combines typed sample-level extraction with GSE and sibling-sample context to resolve ambiguous, incomplete or study-specific annotations, followed by controlled-vocabulary normalization and explicit out-of-vocabulary handling.

LLMs provide a complementary approach because they can integrate distributed textual evidence at inference time (Brown et al. 2020, Dagdelen et al. 2024). In the GEO setting, this matters because a sample annotation may be ambiguous in isolation but interpretable when combined with the GSE title, summary, overall design, and sibling-sample label distribution. Recent biological metadata studies further suggest that language models can help standardize researcher-specific labels and extract biological terms from heterogeneous sample metadata (Ikeda et al. 2025, Verbitsky et al. 2025). Fine-tuned GPT models can assign ontology identifiers to biosample labels with high recall but modest precision (Riquelme-García et al. 2025), and GPT-4o annotation of cell lines and mouse strains in GEO approaches curator performance while still requiring human oversight (Rogic et al. 2026), but both rely on commercial APIs and per-study annotation rather than local, corpus-scale normalization. The key methodological challenge is to exploit this context without over-inferring from the study topic. A pipeline must distinguish the condition of a sample from the disease focus of the study, preserve explicit control and healthy states (e.g., treat “Alzheimer’s control” fundamentally differently from “Alzheimer’s patient”, despite being tagged with the same disease name), and avoid treating generic laboratory protocols as biological treatments.

Here we present a locally deployable LLM pipeline for structured GEO metadata annotation. The pipeline extracts three sample-level fields, Tissue, Condition, and Treatment, together with Sex and Age. It infers selected missing or incomplete labels using study-level context, and links raw labels to MeSH descriptors (Lipscomb 2000) and Cellosaurus cell lines with explicit out-of-vocabulary (OOV) concept handling. Thus, the contribution is not merely the use of an LLM, but the combination of sample-level extraction, study-level contextual inference, and controlled-vocabulary entity linking in a reproducible software workflow.

## 2 Materials and methods

### 2.1 Data sources and benchmark construction

The input data consists of GEO sample records (GSMs) and their associated series-level records (GSEs). For each GSM, the pipeline uses the sample title, source name, characteristics fields, treatment protocol, and description when available. For each GSE, the pipeline uses the title, summary, and overall design to provide study-level context for samples whose fields are incomplete or ambiguous. We use a 1,500-sample disease-focused subset derived from CREEDS (Wang et al. 2016) for evaluation.

CREEDS supports disease-focused development but not GEO-wide claims. Final evaluation therefore uses all applicable ALE-curated Sex and Age values in the final corpus (Giles et al. 2017), plus 1,500-sample Tissue and Condition benchmarks from CREEDS and a 1,500-sample Treatment benchmark adjudicated against GSM/GSE metadata (Section 2.8).

### 2.2 Annotation targets

The pipeline produces three primary labels for each sample, based on its metadata. Tissue denotes the biological source of the sample, including organ, tissue, cell type, cell line, biospecimen, or relevant anatomical site. Condition denotes the disease, phenotype, pathological state, disease stage, or explicit healthy, normal, control, unaffected, or non-disease state that applies to the sample. Treatment denotes the experimental or clinical intervention applied to the sample, including drugs, compounds, genetic perturbations, environmental exposures, irradiation, vehicle controls, sham procedures, and untreated conditions when those are explicit sample states. Sex and Age are also extracted, and are carried through without vocabulary mapping (Fig. 1).

For each field, the pipeline distinguishes between the raw extracted span and the normalized output. The raw span preserves the depositor-provided wording when possible. The normalized output maps the span to a canonical MeSH descriptor when MeSH coverage is appropriate. Concepts without satisfactory MeSH coverage are assigned to local out-of-vocabulary (OOV) concept clusters rather than being forced into an inappropriate MeSH term. No metadata schema is proposed or replaced here: the released label columns are typed by identifier and can be serialised into any container without loss.

### 2.3 Pipeline overview

The pipeline has three conceptual stages (Fig. 1). Phase 1 performs sample-level extraction of Sex, Age, Tissue, Condition, and Treatment. Phase 1b infers selected labels initially marked “Not Specified” or structurally incomplete using GSE-level context and sibling-sample label distributions. Phase 2 links extracted labels to Medical Subject Headings (MeSH) descriptors and Cellosaurus cell-line identifiers through a controlled-vocabulary cascade with decision caching, field-scope enforcement, and out-of-vocabulary (OOV) concept clustering.

### 2.4 Phase 1: sample-level extraction

Phase 1 uses field-specific LLM prompts to extract one or more candidate labels from each GSM for Sex, Age, Tissue, Condition, and Treatment. The Tissue extractor prioritizes explicit cell-line, cell-type, tissue, organ, and source fields. The Condition extractor identifies disease names, phenotypes, stage/grade, and explicit healthy/control states. And the Treatment extractor identifies drugs, doses, vehicles, genetic perturbations, and clinical interventions while excluding sample-handling steps such as RNA extraction. Extractors are run with deterministic decoding and versioned prompts, and return “Not Specified” if a field has no supporting evidence rather than inferring from external knowledge.

### 2.5 Phase 1b: Experiment-level (GSE) inheritance of missing or incomplete sample (GSM) labels

Phase 1b addresses records whose metadata is insufficient in isolation by re-calling the LLM with the parent GSE title, summary, and design, together with the distribution of sibling-sample labels. It recovers labels encoded by study-specific groups or legends, such as treatment arms or disease/control group labels, when that mapping is present in the GSE metadata. We included a safeguard to constrain inference to sample-specific evidence: The GSE topic alone is not treated as evidence that every sample has the disease or received the treatment, and explicit healthy, control, vehicle, sham, or untreated states are preserved. GSE context fills values left Not Specified at the sample level and may also refine an existing one, replacing a study-specific code or an ambiguous span with the concept the study describes. The prompt instructs the model to keep an explicit sample-level value wherever it is already specific, but this rule is stated in the prompt rather than enforced by the software. For Tissue, Condition and Treatment, Phase 1b never demotes a value to Not Specified. Sex normalization produced nine present-to-Not-Specified validator transitions. If the study compares multiple tissues or treatments and the sample is unspecified, the output remains “Not Specified”.

### 2.6 Phase 2: entity linking to MeSH and out-of-vocabulary concept handling

Phase 2 is entity linking, also called concept normalization (Table S34). Phase 2 converts each raw label into a canonical controlled-vocabulary label through an ordered cascade with persistent decision caching (Table S34). For each value it applies, in order: a cache of prior resolutions; a polarity gate; a Cellosaurus match with an LLM cell-line guard (Tissue only); exact MeSH matching; lookup against existing out-of-vocabulary (OOV) concept clusters; per-study expansion of ambiguous short forms followed by re-normalization; BioLORD-2023 retrieval (Remy et al. 2024) with a constrained picker and keep/reject verifier; and, when no vocabulary term faithfully represents the label, minting of an out-of-vocabulary concept. The first stage that resolves a value determines its label. Field scope is enforced throughout: Tissue to anatomy, cell or cell-line concepts (MeSH descriptors and Cellosaurus (Bairoch 2018) identifiers), Condition to diseases and Treatment to drugs, biologics or therapeutic procedures, with labels filed under the wrong field removed from it, diseases out of Tissue and cell lines out of Condition and Treatment (23,482 samples), and those with an unambiguous target moved to the correct field (176 samples). In every removal, the field that should hold the concept already had a label, so no sample was left unlabeled by the step (Table S22). OOV concepts are then consolidated - surface-form variants are shortlisted by BioLORD-2023 similarity (0.90 cosine floor) and merged under an LLM decision, under a conservative merge rule designed to avoid collapsing distinct concepts. Two deterministic steps close the phase: a case/whitespace/hyphen fold onto a single canonical identifier, and a within-sample de-duplication that collapses repeated identical ids arising when two spans of one sample denote the same concept, preserving order and leaving distinct ids and ‘Not Specified’ untouched. The picker chooses among retrieved candidates by index and never writes an identifier itself, so a value it declines is left unresolved rather than assigned a term. All per-component prompts are listed in the Supplement.

### 2.7 Local deployment, checkpoints, and reproducibility

Hardware and serving. Phase 1 (sample-level extraction and GSE-context inference of Sex, Tissue, Condition and Treatment) was served by google/gemma-4–12B-it (Gemma Team 2026) with fp8 weight quantization and a 16-bit KV cache (8,192-token context) on an 8-GPU fp8-capable node, one model instance per GPU. Age extraction and Phase 2 entity linking were served by google/gemma-4-E2B-it in bf16 on a single node of 8x NVIDIA GeForce RTX 3090, one model instance per GPU under vLLM (Kwon et al. 2023). BioLORD-2023 candidate embeddings were computed on CPU. For Phase 2 and Age the model was run as a text-only derivative of this natively multimodal model, retaining only its 601 text-decoder tensors; text-generation outputs are identical.

All model weights, MeSH/Cellosaurus databases, embedding indexes, decision caches, and per-GSE checkpoints reside on user-controlled hardware. The only optional network access fetches missing GSE-level metadata from NCBI GEO. Reproducibility is supported through versioned prompts, logged model and backend configuration, deterministic decoding where supported, per-GSE checkpoints, and audit logs for LLM calls; bit-level reproducibility is not guaranteed across GPU architectures, quantization, serving engines, or parallel schedules, so exact software versions, hardware, model checkpoints, and cache state are archived with each benchmark run. The pipeline is packaged for reuse. A single-workstation configuration and a multi-GPU cluster configuration share the same prompts, databases, and runners, so other users can reproduce the benchmark or apply the pipeline to a new corpus by changing the input path, without code changes.

### 2.8 Evaluation design

Phase 1 is evaluated without an automated judge. Sex and Age use all applicable ALE-curated records in the final corpus (n = 9,299 and 5,164). Each sample contributes one outcome, partitioned into true positives, substitutions, false alarms, false negatives and true negatives, so the categories sum to n (Table S1). Multi-label cells are scored in Table S31. Tissue and Condition use 1,500 CREEDS samples matched by concept, synonym, morphology and hierarchy, and Treatment uses 1,500 samples adjudicated against GSM/GSE metadata. Tissue, Condition and Treatment benchmark verdicts were assigned manually. Confidence intervals use Wilson’s method (Wilson 1927); because samples from one study are not independent, cluster-bootstrap intervals and per-study, per-concept and micro- and macro-averaged figures are given in Tables S27-S29. Phase 2 is evaluated deterministically on labels with an exact in-scope MeSH or Cellosaurus answer. A wrong database ID counts as FP, and an OOV or unresolved result with no database identifier counts as FN. Corpus-integrity audits and the LLM-judged OOV and multilabel analyses are supplementary evaluations, not substitutes for these gold-standard benchmarks (Tables S1, S5-S7 and S18).

### 2.9 Metrics

Phase 1 extraction is reported as precision, recall and F1 per field (Table 1). Phase 2 entity linking is reported as database-resolution precision, recall, F1 and accuracy on labels with an unambiguous vocabulary answer, with explicit counts of false matches and missed resolutions (Table 2), per-vocabulary match precision, the inappropriate-OOV rate, fragmentation-collapse and consolidation counts (Table 3). Short-form expansion accuracy is verified against each study’s own description. MeSH branch validity and identical-input consistency are reported at 100% corpus coverage. Supplementary Tables S1-S35 give the per-table breakdowns. Table S7 reports the independent LLM-judge audit of out-of-vocabulary completeness (16.29% inappropriate-OOV among 1,915 decided audit items, 95% CI 14.71–18.01, not a design-weighted population estimate). Two supplementary notes report multi-label precision and the per-field error analysis. All deterministic metrics are computed at 100% coverage and are reproducible from the released data.

## 3 Results

The main expected benefit of Phase 1b is recovery of labels that are Not Specified or incomplete at the GSM level but explicitly resolvable from GSE context. Using GSE context, gemma-4–12B-it recovered 76.58% of Not-Specified Tissue values, 45.63% of Condition, and 10.89% of Treatment, raising coverage to 99.74%, 80.43%, and 45.91% respectively. Sex receives no GSE-context recovery and is finalized at Phase 1b. The three phases are set side by side per label in Table S35. Age is produced by a separate reasoning-enabled gemma-4-E2B-it pass (Supplementary Table S3).

### 3.1 Controlled-vocabulary normalization reduces label fragmentation

Across all 804,427 samples, Phase 2 collapsed 21,254 distinct raw Tissue strings to 10,548 canonical concepts, 21,808 Condition strings to 15,679, and 48,216 Treatment strings to 40,287 (Table 3). In total 26,388 distinct labels resolved to MeSH descriptors and 6,340 Tissue labels to Cellosaurus identifiers (e.g. MCF7→CVCL_0031), and the remainder were assigned to out-of-vocabulary concept clusters that preserve specificity rather than being forced onto an incorrect MeSH hypernym. Those raw values collapse onto 57,846 distinct concepts: 3,441 MeSH descriptors, 2,599 Cellosaurus cell lines and 51,806 out-of-vocabulary concepts, spanning 482,725 samples with a MeSH tissue, 166,041 with a named cell line and 407,126 with a MeSH disease (Table S21). A further 5,420 out-of-vocabulary variants were consolidated into shared concepts (e.g. there were 19 different spellings of “CD4 + T cells”; Table S8), and a final deterministic fold unified 268 further fold-groups covering 22,916 samples, of which 4,177 changed identifier (Table S12) (PBMC and pbmc, wild type and wildtype now share one identifier). Biologically distinct states (control, healthy, normal) and marker variants (CD4 vs CD4+) are deliberately kept separate. A deterministic MeSH-branch check found violation rates of 0.00% (Tissue), 0.44% (Condition) and 2.07% (Treatment) (Table 3, Table S33). Identical inputs do not always receive the same target (context-free identical-input disagreement of 18.84%/21.97%/16.96%; Table S33). On the in-dictionary subset (a silver standard; 342,821/219,651/23,261 instances) resolution reached F1 0.993, 0.996 and 0.991 with 4,246, 1,225 and 271 false matches (Table 2). Accuracy over the full label distribution is anchored by the manual gold (Table 1). Study-level context correctly expanded ambiguous short forms within their studies (COPD→Pulmonary Disease, Chronic Obstructive; MS→Multiple Sclerosis), verified against each study’s own description (Table S9). Compared with deterministic exact MeSH matching, the pipeline assigned a controlled-vocabulary identifier to a larger fraction of extracted labels in every field (Table S13). The per-field error analysis and the multi-label audit are given in the two supplementary notes. Per-study, per-concept and metadata-grounding breakdowns of these scores are in Tables S27, S29 and S30.

### 3.2 Internal consistency checks of the annotations

Benchmark metrics cannot detect systematic errors outside the benchmark’s scope. We therefore conducted a few “sanity checks” by searching for label combinations that should not occur (Table 4). These are raw contradiction rates between two nominal classifications of the same samples rather than chance-corrected agreement (Cohen 1960), because one side of each compariso is a deterministic vocabulary lookup and not an independent rater.

For the disease-age check, Alzheimer samples showed no violations (0 of 1,286), and 333 of the 348 violations came from COPD samples in longitudinal studies, where a follow-up timepoint recorded under an age-named field was captured as the subject’s age (e.g. ‘age: M3’ - study month 3, or ‘days: 84’ where 84 is the number of days into the study).

## 4 Discussion

We developed a locally deployable LLM pipeline for extracting and normalizing structured biological labels from heterogeneous GEO sample metadata. Useful sample-level annotations often require both local textual extraction and controlled use of study-level context. By separating sample-level extraction, context inference, and entity linking to MeSH, the pipeline provides an auditable route from depositor-written metadata to analysis-ready labels. The most important practical implication is improved reuse of public transcriptomic data. Harmonized Tissue, Condition, and Treatment labels enable disease-level meta-analysis, tissue-stratified cohort construction, perturbation signature aggregation, and better filtering of samples for downstream analyses. These labels may also support metadata conditioning for transcriptomic foundation models and quality control of large public compendia.

The study-context inference stage addresses a common limitation of sample-level metadata: a GSM record is often not self-contained, since group codes, treatment arms or disease/control definitions may only be interpretable from the GSE design. This same feature creates the main risk of LLM-based annotation, over-inference. The pipeline therefore treats explicit sample-specific evidence as primary and uses GSE context only when it resolves ambiguity without contradicting the sample record. Controlled-vocabulary entity linking is necessary because extraction alone leaves labels fragmented across synonyms, abbreviations, spelling variants and study-specific phrasing. MeSH is a useful target for many diseases, tissues and treatment classes but does not cover all experimental constructs, engineered perturbations or cell-line variants; out-of-vocabulary clustering is therefore a safeguard against forcing incorrect mappings.

Local execution is another important feature. Running open-weight models on local hardware avoids dependence on external LLM APIs, improves auditability, and allows versioned prompts, caches, checkpoints and outputs to be archived. LLM reproducibility nevertheless remains imperfect across model versions, quantization settings, hardware and serving backends, so the release pins model identifiers and dependencies and provides the archived per-sample outputs together with scripts that recompute the principal deterministic metrics. The reproducible comparison in Table S13 is assembled from those deterministic outputs. The complete pipeline, including an interactive interface ready to annotate further GEO samples, is available at https://github.com/SciSpectator/LLM-GEO-Label-Extractor and runs the three stages end-to-end.

This work is complementary to existing GEO and metadata resources. GEOmetadb improves structured access to GEO metadata (Zhu et al. 2008), MetaSRA provides normalized sample annotations for SRA (Bernstein et al. 2017), and CREEDS provides curated disease signatures (Wang et al. 2016). The present pipeline targets a different but related problem: extracting and normalizing structured labels from heterogeneous GEO sample-level text using contextual evidence and local LLM inference.

### 4.1 Limitations

This study has several limitations. First, performance estimates depend on the benchmark: a disease-focused CREEDS subset doesn’t establish general performance across all GEO study types. Second, Treatment annotation is difficult because the evidence may reside in protocol text, group labels or series design, and no curated resource provides comprehensive treatment gold labels. Third, MeSH is limited in the concepts it covers. Fourth, we provided very limited semantic normalization to the labels (e.g., control, healthy and normal are conceptually similar but not merged; marker variants such as CD4 and CD4 + are not merged). Finally, the database-resolution F1 in Table 2 measures controlled-vocabulary matching only for labels with a MeSH or Cellosaurus answer. Beyond that bound, an independent LLM judge, Claude Opus 5 (Anthropic 2026), a different model family from the pipeline was used.

## Supplementary Files

This is a list of supplementary files associated with this preprint. Click to download.

- SupplementaryMSzczepaniakJWren.docx

## Figures and Tables

**Figure 1:**
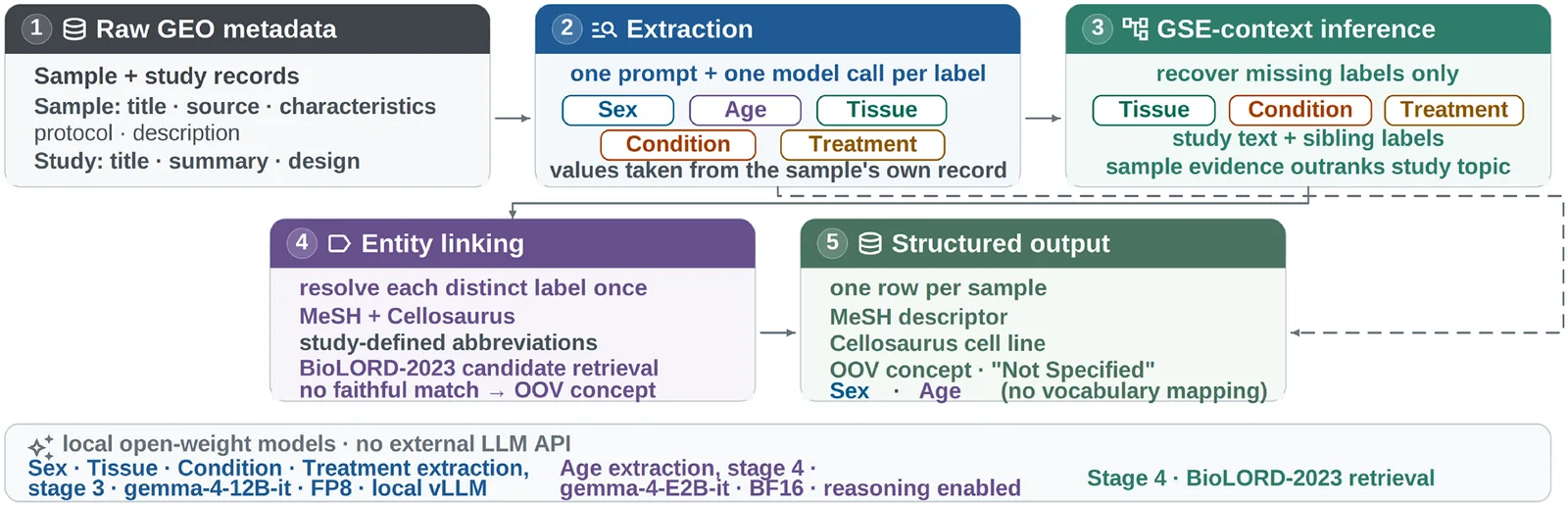
Conceptual workflow. Sample and study records enter an extraction stage that returns five labels independently, one prompt and one model call per label, taking only values supported by the sample’s own record. Only Tissue, Condition and Treatment continue: unspecified values are recovered from study text and sibling labels, sample evidence outranking the study topic. Sex and Age bypass both later stages (dashed) and are carried through unmapped. Entity linking resolves each distinct value once against MeSHand Cellosaurus, expanding abbreviations from the defining study and using BioLORD-2023to propose candidates, and assigns an out-of-vocabulary concept where none is faithful. Output is one row per sample.

**Table 1:** Phase 1 extraction, in two separate tables so that no row joins a corpus count to a benchmark score. Left: how many of the 804,427 samples carry a label after Phase 1b. Right: how accurate those labels are, each row measured only on the benchmark whose size is given in the same row (Table S1). Study-level and concept-level aggregates are in Tables S27-S29.

Field	Labels extracted	% of 804,427		Field	Benchmark n	Precision	Recall	F1
Sex	319,745	39.75%		Sex	9,299	0.997	0.623	0.767
Age	383,173	47.63%		Age	5,164	0.987	0.976	0.982
Tissue	802,359	99.74%		Tissue	1,500	0.997	0.997	0.997
Condition	647,007	80.43%		Condition	1,500	0.964	0.950	0.957
Treatment	369,311	45.91%		Treatment	1,500	0.976	0.847	0.907

**Table 2:** Phase 2 database resolution on the exact-name/synonym in-dictionary subset. A wrong database ID counts as FP, an answerable label left OOV or unresolved counts as FN. Accuracy is TP divided by gold-positive answerable items. Full definitions and audits are in Tables S5-S7 and S18.

Field	Labels w/DB answer	Precision	Recall	F1	Accuracy	Wrong DB ID	No DB ID (OOV/unresolved)
Tissue	342,821	0.988	0.998	0.993	0.985	4,246	791
Condition	219,651	0.994	0.998	0.996	0.993	1,225	382
Treatment	23,261	0.988	0.994	0.991	0.982	271	149

**Table 3:** Whole-corpus normalization summary (804,427 samples, distinct-value counts). “→**”** means “routed to”. Branch violations = MeSH assignments outside the field’s tree branch.

Field	# unique label values	# canonical	→ MeSH	→ Cellosaurus	→ OOV	Uncovered	Branch viol.
Tissue	21,254	10,548	8,068	6,340	6,682	164	0.00%
Condition	21,808	15,679	11,332	n/a	9,148	1,328	0.44%
Treatment	48,216	40,287	6,988	n/a	39,201	2,027	2.07%

**Table 4:** Sanity checks on the released outputs (804,427 samples), reported as found - that is, on the first-pass labels, before the corrective re-extraction described in the text. n comparable = assignments the screen can evaluate.

Field checked	Checked against	n comparable	Contradictions (rate)
Extracted Sex	Sex-specific tissue	9,522	11 (0.12%)
Extracted Sex	Sex-specific condition	3,761	55 (1.46%)
Extracted Sex	Cell-line donor sex	79,551	571 (0.72%)
Corpus species	Cell-line species	167,476	1,076 (0.64%)
Extracted age	Late-onset disease (AD, PD & COPD with age < 18 years)	4,102	348 (8.48%)

## Data Availability

All code, evaluation scripts and per-sample results for the 804,427 samples are provided as Supplementary Data, including the full ALE-overlap Sex/Age evaluation, the 1,500-sample Tissue/Condition/Treatment benchmarks with verdicts manually assigned by MS without an LLM judge, and the scripts that recompute Table 1. An interactive program built from this pipeline, ready to annotate further GEO samples, is available at https://github.com/SciSpectator/LLM-GEO-Label-Extractor. Model identifiers, decoding settings and pinned dependencies are listed in the Supplementary Data.
